# The first three years of the *Journal of Global Health*: Assessing the impact

**DOI:** 10.7189/jogh.04.010101

**Published:** 2014-06

**Authors:** Igor Rudan, Harry Campbell, Ana Marušić

**Affiliations:** 1 Centre for Population Health Sciences and Global Health Academy, University of Edinburgh Medical School, Scotland, UK; 2 Croatian Centre for Global Health, University of Split School of Medicine, Split, Croatia; 3 Department of Research in Biomedicine and Health, University of Split School of Medicine, Split, Croatia

## Abstract

The *Journal of Global Health* (JoGH) is three years old. To assess its impact, we analysed online access to JoGH's articles using PubMed Central and Google Analytics tools. Moreover, we tracked citations that JoGH received in 2013 using ISI Web of Knowledge^SM^ and Google Scholar® tools. The 66 items (articles, viewpoints and editorials) published between June 2011 and December 2013 were accessed more than 50 000 times during 2013, from more than 160 countries of the world. Seven among the 13 most accessed papers were focused on global, regional and national epidemiological estimates of important infectious diseases. JoGH articles published in 2011 and 2012 received 77 citations in Journal Citation Reports® (JCR)–indexed journals in 2013 to 24 original research articles, setting our first, unofficial impact factor at 3.208. In addition, JoGH received 11 citations during 2013 to its 12 original research papers published during 2013, resulting in an immediacy index of 0.917. The number of external, non–commissioned submissions that we consider to be of high quality is continuously increasing, leading to current JoGH's rejection rate of about 80%. The current citation analysis raises favourable expectations for the JoGH's overall impact on the global health community in future years.

On March 7, 2014, the *Journal of Global Health* (JoGH) was exactly three years old. We published the first three volumes, delivering all on time – both electronically and in print, while adhering closely to our initial concept of the journal content: an editorial followed by a comprehensive summary of global health news, four viewpoints and six articles. We have every reason to celebrate our journal's third birthday, because this is perhaps the most significant milestone in the life of a new journal: the first opportunity when its impact on the wider scientific community can be adequately assessed.

In the not–so–recent–past, when manuscripts were still being submitted in heavy paper envelopes by regular mail, typed double–spaced and photocopied in triplicate, with figures often drawn by hand, and with travel times across the Atlantic of three weeks in each direction, the only way to evaluate the impact of a start–up journal was through the subscriptions and citations that it would attract. Subscriptions to printed copies were almost a necessity, providing funds needed to ensure journal's sustainability, which is why most journals arose from professional societies. The search for citations was done by painstaking browsing through heavy, voluminous books that resembled phone directories of large cities – month by month, author by author, article by article. That was the reality of scientific publishing and impact assessment that had remained fairly unchanged throughout most of the 20^th^ century.

The three JoGH’s Editors–in–Chief still remember those times very well, both as authors of manuscripts and as journal editors. However, we are also aware that researchers who started their careers in the 21^st^ century probably cannot even begin to comprehend those times. Article mailing charges could have cost researchers from low–income countries their week's salary, which they were willing to pay despite an uncertain outcome. This obstacle is now replaced in most journals with a faceless electronic submission and electronically generated e–mail replies. The model of financing through subscriptions has been largely replaced, too. Many journals charge access to research articles that they publish. The traditional, reader–pays publishing model is today challenged by the open–access movement. New open–access journals are being launched on an almost daily basis, offering free access to their content, but charging authors for the article processing and publishing costs. Cyberspace has entirely replaced print, and it is difficult for us to remember holding a printed issue of any journal in our hands and reading it, article–by–article, as it was done only a decade ago. Nowadays, PDF versions of individual articles are downloaded, searched using browsers, and stored somewhere in the computer for further reading.

In this sea of change that has completely transformed scientific publishing, there is still one surviving feature that seems fitter than ever: the almighty “impact factor”. Although much criticized, terribly flawed in so many ways, calculated in a non–transparent way, generated by a single, now private, enterprise, with numerator and denominator often not containing comparable items, and having a long history of being manipulated to a greater or lesser extent – it still remains the single most effective advertisement for any scientific journal, dwarfing all others by a large margin [1–3]. Therefore, anyone serious about their scientific publishing effort – and we certainly aim to be – simply cannot afford to ignore it, no matter what we may think of it personally.

The impact factor (IF) was instantly and firmly accepted by the scientific community because is successfully reduced all information about a journal’s content to a single number. For the vast majority of journals, their IF ranges between 0 and 10: among 8471 journals included in the Journal Citation Reports® Science Edition in 2012, which are themselves considered to represent a selection of the world's journals of the highest quality, 8312 (98.1%) had IF smaller than 10 [4]. This means that only a small minority of the most competitive journals have the IF greater than 10. The IF tells any interested researcher the average number of citations that the articles published by the journal over the previous two calendar years received in the current calendar year. Subsequently, this implies that any articles that attracted 10 or more citations in any calendar year generated a substantial interest in the research community.

There have been developments in recent years that promise to at least provide some validation for the calculated impact factors, if not actually offering a viable alternative. First, the ISI Web of Knowledge^SM^ Journal Citation Reports® (JCR) by Thomson Reuters publishing corporation provides impact factors based both on 2–year and 5–year content follow–up, which prevents and exposes manipulation of the original 2–year metric [4]. Moreover, they also provide the Eigenfactor® score and Article Influence® score. The Eigenfactor® score calculation is based on the number of times that the articles from the journal, that were published in the previous five years, have been cited in the current year, but it also takes into account the quality of the journals that have contributed these citations (ie, citation in a journal with a higher impact factor will influence this score more than one in a journal with a lower impact factor) and it removes a journal's self–citations [4]. The Article Influence® score determines the average influence of a journal's articles over the first five years after publication. It is calculated by dividing a journal’s Eigenfactor® score by the number of articles in the journal, normalized as a fraction of all articles in all publications. The mean Article Influence® score is 1.00, and a score greater than 1.00 indicates that each article in the journal has above–average influence, and vice versa [4]. These additional metrics contribute additional validation to the original impact factor alone.

However, this still doesn't address the concern that all measurement of quality of scientific journals seems to be in hands of a single, private enterprise, and is dependent on their choice of the journals that are being followed and that contribute citations. They also make decisions on how to classify journal's published items, which determines the denominator of the IF equation. However, things have changed in this area, too, and competition has emerged. The ISI Web of Knowledge^SM^ searching tool– Web of Science^TM^ [5] – is no longer the only prominent web–based provider of citations to the published articles. Another publishing giant, the Reed Elsevier corporation, have developed their own citation database – Scopus® [6]. It is a very similar search tool, although possibly more comprehensive in some areas of science – it covers more than 20 000 titles from over 5000 publishers, offering about 20% more coverage than Web of Science^TM^ [7]. Both of these search engines require relatively expensive subscription for access. However, Google, Inc. corporation, which states that their mission is “...*to organise the world’s information and make it universally accessible and useful*”, have launched their own, free search engine that also tracks citations – Google Scholar® [8]. The coverage of journals and academic sources in Google Scholar is not only completely free to the general public, but also much more comprehensive than either Web of Science® or Scopus®. This is because it takes into account citations found in virtually any document that has ever been exposed to the internet, in any shape or form. Therefore, citations to published articles can nowadays be tracked using at least three tools – Web of Science®, Scopus® and Google Scholar®. They will quote different number of citations, with the first two being less inclusive, and the third one being more inclusive.

In addition to improved tracking of article citations and journal's impact, another metrics of scientific impact has emerged – Hirsch index (or h–index) [9]. Designed initially to capture the productivity of any individual scientists in a single number, this metric can also be applied to scientific journals. h–index measures the number of articles associated with a scientist, or a journal – *h* – that have been cited *h* times or more. That means that a scientist, or a journal, with h–index of 50 would have published 50 articles that have each been cited 50 times or more. All other articles associated with this scientist would have been cited less than 50 times, and therefore they would not contribute to the score. The beautiful simplicity of this metric and its ability to capture both the quality and the quantity of research output in a single number has made it extremely popular in recent years [10]. Web of Science®, Scopus® and Google Scholar® also provide it in association to research output of individual scientists. Interestingly, Google Scholar recognised its value in evaluating scientific journals, too, and it provides ranking of world's leading 100 journals in different languages by their 5–year h–index, ie, the h–index based on citations to all papers published in the previous 5 years [9]. While impact factor favours journals that publish small, selected number of papers which attract high number of citations each year (such as journals that specialize in publishing review articles), 5–year h–index does more justice to journals that publish large number of quality papers. With h–index, the quantity of published papers becomes a potential strength, as more papers could contribute to h–index; while with impact factor publishing many papers could be seen as a burden to achieving high metric, because they increase the denominator.

Finally, the widespread use of the internet and social media, and the possibility to document and store the information about its usage, has lead to an entirely new way of evaluating the impact of scientific publishing. Given that the papers are now accessed on the Internet rather than by reading printed journals, this allowed evaluation of the impact not only through article, but also through their access and usage. Thanks to tools such as PubMed Central® [11] and Google Analytics® [12], it is possible to follow access and downloads of individual articles in their electronic or PDF forms, tracing them to geographic location and other characteristics of the user. This has shown that many articles, especially those related to policy, are used, read and commented lot more than they are cited. Social media such as Facebook [13] and Twitter [14] allow following of how much immediate impact do research articles generate, and how quickly do their ideas spread through social media – ie, how often are they “shared”, “tweeted” and “liked”. This allowed a broad, multi–dimensional evaluation of research impact that could not even have been imagined only a decade ago.

So, finally to the point – how did the *Journal of Global Health* do over the past three years, taking into account all those different measures of scientific impact that exist today? We decided to focus on two measures of usage and two citation–tracking tools. The measures of usage are based on online access to the articles through PubMed Central [11] and through our own journal's website, monitored using Google Analytics tools [12]. The two citation–tracking tools were ISI Web of Knowledge^SM^ [5] and Google Scholar® [8].

**Table 1** shows the ranking (according to total access, ie, usage) of the 20 most accessed papers published in the first 3 volumes – between June 2011 and December 2013. The 66 items (articles, viewpoints and editorials) published during this period were accessed more than 50 000 times and from more than 160 countries of the world. **Table 1** shows that among all recorded episodes of access, full–text access was typically 2–4 times more common than PDF download. Also, the access occurred about 8 times more frequently through PubMed Central than through our own website, as a result of PubMed searches that pointed to our content.

**Table 1:** Ranking the 20 most accessed papers published by the *Journal of Global Health* in the first 3 volumes (between June 2011 and December 2013).

Rank	Author	Title	Citation	Total requests	Full text requests	PDF requests	Citations (WoK)	Citations (Google Scholar)
1	Igor Rudan et al; CHERG group	Epidemiology and etiology of childhood pneumonia in 2010: estimates of incidence, severe morbidity, mortality, underlying risk factors and causative pathogens for 192 countries	J Glob Health 2013; 3(1):010401	3718	2387	1331	5	9
2	Geoffrey C. Buckle et al.	Typhoid fever and paratyphoid fever: Systematic review to estimate global morbidity and mortality for 2010	J Glob Health. 2012; 2(1):010401	2350	1763	587	13	20
3	Jacqueline Torti	Floods in Southeast Asia: A health priority	J Glob Health. 2012; 2(2):020304	2082	1964	118	1	1
4	Issrah Jawad et al.	Assessing available information on the burden of sepsis: global estimates of incidence, prevalence and mortality	J Glob Health. 2012; 2(1):010404	1814	1298	516	4	10
5	MahbubaMeem et al.	Biomarkers for diagnosis of neonatal infections: A systematic analysis of their potential as a point–of–care diagnostics	J Glob Health. 2011; 1(2):201–209	1798	1098	700	4	6
6	David Hipgrave	Communicable disease control in China: From Mao to now	J Glob Health. 2011; 1(2):224–238	1424	1179	245	7	10
7	Ivana Kolčić	Double burden of malnutrition: A silent driver of double burden of disease in low and middleincome countries	J Glob Health. 2012; 2(2):020303	1243	999	244	2	4
8	Donald Waters et al.	Aetiology of community–acquired neonatal sepsis in low and middle income countries	J Glob Health. 2011; 1(2):154–170	1171	850	321	2	4
9	Tom K. Roberts et al.	Epidemiology and aetiology of maternal parasitic infections in low– and middle–income countries	J Glob Health. 2011; 1(2):189–200	1149	764	385	3	7
10	Rajiv Bahl et al.	Setting research priorities to reduce global mortality from preterm birth and low birth weight by 2015	J Glob Health. 2012; 2(1):010403	1128	745	383	5	8
11	Ruth M. Campbell et al.	The importance of a common global health definition: How Canada's definition influences its strategic direction in global health	J Glob Health. 2012; 2(1):010301	1080	899	181	2	3
12	Shelby E. Wilson et al.	Scaling up access to oral rehydration solution for diarrhea: Learning from historical experience in low and high–performing countries	J Glob Health. 2013; 3(1):010404	1006	808	198	1	4
13	Prasad Palani Velu et al.	Epidemiology and aetiology of maternal bacterial and viral infections in low– and middle–income countries	J Glob Health. 2011; 1(2):171–188	988	761	227	2	3
14	Harish Nair and Rajmohan Panda	Quality of maternal health care in India: Has the National Rural Health Mission made a difference?	J Glob Health. 2011; 1(1):79–86	952	818	134	2	2
15	Anthony Maher and Devi Sridhar	Political priority in the global fight against non–communicable diseases	J Glob Health. 2012; 2(2):020403	911	610	301	5	7
16	Rhiannon George–Carey et al.	An estimate of the prevalence of dementia in Africa: A systematic analysis	J Glob Health. 2012; 2(2):020401	907	611	296	2	3
17	Xing Lin Feng et al.	Social, economic, political and health system and program determinants of child mortality reduction in China between 1990 and 2006: A systematic analysis	J Glob Health. 2012; 2(1):010405	723	520	203	6	9
18	Igor Rudan et al.	Setting priorities for development of emerging interventions against childhood pneumonia, meningitis and influenza.	J Glob Health. 2012;2(1):010304	713	417	296	5	10
19	Thor A. Wagner et al.	Emerging biomarkers for the diagnosis of severe neonatal infections applicable to low resource settings	J Glob Health. 2011; 1(2):210–223	709	532	177	3	4
20	Alice Graham et al.	Estimating the incidence of colorectal cancer in Sub–Saharan Africa: A systematic analysis	J Glob Health. 2012; 2(2):020404	709	491	218	3	4

WoK – Web of Knowledge

Seven among the 13 most accessed papers were focused on global, regional and national epidemiological estimates of important infectious diseases: childhood pneumonia (ranked 1^st^), typhoid and paratyphoid fever (2^nd^), sepsis (4^th^), neonatal sepsis (8^th^), maternal parasitic infections (9^th^), childhood diarrhoea (12^th^) and maternal bacterial and viral infections (13^th^). The high position of those articles could have been somewhat expected, but the most pleasant surprises on the list were the articles on the floods in Southeast Asia as a health priority (3^rd^), biomarkers for neonatal sepsis (5^th^), a historical perspective on communicable disease control in China (6^th^) and malnutrition as a contributor to “double burden of disease” in poor countries (7^th^). Two further related clusters worth mentioning were three papers on the topic of non–communicable diseases in low– and middle–income countries (ranked 15^th^, 16^th^ and 20^th^), and the two research priority–setting exercises that used the CHNRI method (ranked 10^th^ and 18^th^).

**Table 1** also shows that access indicators correspond reasonably well to the number of citations received, especially when analyzed across all 66 published items and normalized for the number of months since the time of the publication. The number of citations recorded by Google Scholar® was typically between 25% and 75% larger than the number recorded by ISI Web of Knowledge^SM^. The two most accessed papers also stand out in terms of the number of citations received (especially when this is adjusted for the duration of citing period), but several other papers are already showing that they will likely accumulate at least 10 citations in ISI Web of Knowledge^SM^ during 2014.

Using the citation data from Google Scholar, we calculated JoGH’s impact factor: we added the number of all citations received during 2013 from the journals that are indexed by the ISI Web of Knowledge^SM^, and then divided the sum by the number of our “citeable” items published in 2011 and 2012, ie, original research articles published in JoGH (because our viewpoints are published as opinion pieces). JoGH articles from 2011 and 2012 received 77 citations in 2013 to 24 original research articles in 2011 and 2012, setting our first, unofficial impact factor at 3.208. In addition, we received 11 citations during 2013 to our 12 original research papers published during 2013, leading to our first immediacy index of 0.917. Our journal's self–citations did not contribute to this calculation because JoGH is still not indexed among the journals that the ISI Web of Knowledge^SM^ and the Journal Citation Reports® use for computing impact factors [15].

Can we be satisfied with our impact to date? The numbers above are certainly encouraging. When we launched the journal in 2011, we defined our mission as serving “*the community of researchers, funding agencies, international organizations, policy–makers and other stakeholders in the field of international health by providing an independent assessment of the key issues that dominated the previous semester in the field of global health and development; publishing high–quality peer–reviewed original research; providing objective reviews of global health and development issues; and allowing independent authors and stakeholders to voice their personal opinions on issues in global health*”. We seem to have addressed all those goals to a substantial extent – through publishing a selection of topical research articles, viewpoints and news items that have been noticed in the global health community. Given that we first appeared on PubMed Central in January 2013, and that the large majority of access to our content is achieved through PubMed searches that return our papers as a result, the total access to our content (more than 50 000 requests) has essentially been achieved over a period of a single year. Moreover, this means that our average published paper has been seen nearly 1000 times over the past year – ie, between 2–3 times each day. In addition, we were pleasantly surprised by the fact that we recorded access to our content from nearly every country in the world over the period of just over a year. This is certainly not a negligible impact. Moreover, the current citation analysis raises favourable expectations for the future years, especially taking into account the average time between access to articles and their citations – suggesting that the potential for translation of a considerable recorded access to our published content into citations is yet to be revealed.

The encouraging signs that our journal is taken increasingly seriously among the major players in global health are reflected in the fact that the number of external, non–commissioned submissions that we consider to be of high quality is continuously increasing: while we only received one external, non–commissioned submission in our first year (June–December 2011), we received 6 external submissions in 2012, then 42 in 2013, while the projection for the 2014 based on the first three months is already above 70. We publish a total of 20 items each year, which means that our rejection rate (when all the commissioned papers are added) is already approaching 80%. Another sign of our increasingly notable presence in the global health research community is reflected in the fact that we already have theme issues produced in collaboration with leading global health organizations – Program for Appropriate Technology in Health (PATH) and The Gates Foundation (2013 June theme issue on childhood diarrhoea), Imperial College London's Global eHealth Unit (2013 December theme issue on mHealth), and United Nation's Children Fund (UNICEF – 2014 December theme issue on integrated community case management), at the same time keeping a rigorous peer review process. We are looking forward to further similar collaborations, as a growing evidence of our journal's increasing impact in global health research community.
